# Single-Cell Profiles of Age-Related Osteoarthritis Uncover Underlying Heterogeneity Associated With Disease Progression

**DOI:** 10.3389/fmolb.2021.748360

**Published:** 2022-01-10

**Authors:** Wenzhou Liu, Yanbo Chen, Gang Zeng, Shuting Yang, Tao Yang, Mengjun Ma, Weidong Song

**Affiliations:** ^1^ Department of Orthopedics, Sun Yat-sen Memorial Hospital, Sun Yat-sen University, Guangzhou, China; ^2^ Department of Anesthesia, Sun Yat-sen Memorial Hospital, Sun Yat-sen University, Guangzhou, China; ^3^ Department of Emergency, Sun Yat-sen Memorial Hospital, Sun Yat-sen University, Guangzhou, China; ^4^ Department of Orthopedics, The Eighth Affiliated Hospital, Sun Yat-sen University, Shenzhen, China

**Keywords:** osteoarthritis, synovial, macrophages, TNF pathway, inflammatory cytokine, cytokine receptor

## Abstract

**Objective:** Osteoarthritis (OA) is the most common chronic degenerative joint disease, which represents the leading cause of age-related disability. Here, this study aimed to depict the intercellular heterogeneity of OA synovial tissues.

**Methods:** Single-cell RNA sequencing (scRNA-seq) data were preprocessed and quality controlled by the Seurat package. Cell cluster was presented and cell types were annotated based on the mRNA expression of corresponding marker genes by the SingleR package. Cell-cell communication was assessed among different cell types. After integrating the GSE55235 and GSE55457 datasets, differentially expressed genes were identified between OA and normal synovial tissues. Then, differentially expressed marker genes were overlapped and their biological functions were analyzed.

**Results:** Totally, five immune cell subpopulations were annotated in OA synovial tissues including macrophages, dendritic cells, T cells, monocytes and B cells. Pseudo-time analysis revealed the underlying evolution process in the inflammatory microenvironment of OA synovial tissue. There was close crosstalk between five cell types according to the ligand-receptor network. The genetic heterogeneity was investigated between OA and normal synovial tissues. Furthermore, functional annotation analysis showed the intercellular heterogeneity across immune cells in OA synovial tissues.

**Conclusion:** This study offered insights into the heterogeneity of OA, which provided in-depth understanding of the transcriptomic diversities within synovial tissue. This transcriptional heterogeneity may improve our understanding on OA pathogenesis and provide potential molecular therapeutic targets for OA.

## Introduction

Osteoarthritis (OA) represents the most frequent age-associated chronic degenerative joint disease (Maumus et al., 2020). Aging is the key driving force in OA (Deng et al., 2019). The incidence of OA in the middle-aged population is 40–80%, and the disability rate is >50% (Zhang et al., 2020b). The diverse aetiology of OA is usually the result of many overlapping factors. The pathogenesis of OA involves the entire joint, composed of articular cartilage, synovial membrane as well as subchondral bone (Griffin and Scanzello, 2019). Traditional therapeutic strategies such as non-steroidal anti-inflammatory drugs only ease pain symptoms. Joint replacement may effectively treat OA patients at the end stage, with relatively high surgery risk and economic cost (Zhou et al., 2020). At current, there is still a lack of FDA-approved disease-modifying OA drugs that may remiss the pain and restrain joint degradation (Grandi and Bhutani, 2020).

Synovitis is common in OA and may augment cartilage damage, which has been a therapeutic target of OA (Labinsky et al., 2020). Synovitis is characterized by synovial tissue hyperplasia, inner macrophage aggregation as well as cell secretion disfunction (Li and Zheng, 2020). The immune system that is involved in the initiation and progression of OA is a key element in the pathogenesis of this disease (Zhang et al., 2019). The inflammatory microenvironment of OA synovial tissues consists of immune cells and inflammatory mediators such as inflammatory cytokines (IL-1β, TNFα, IL-6, IL-15, IL-17, and IL-18) and anti-inflammatory cytokines (IL-4, IL-10, and IL-13) (Wojdasiewicz et al., 2014). The pathophysiological processes that occur in the OA joint are mostly mediated by inflammatory mediators secreted from immune cells. Determining the characteristics of immune cells and mediators in OA synovium may reveal critical features of OA pathogenesis (Zheng et al., 2021). So far, it remains indistinct on the roles of inflammatory mediators in the context of OA synovium.

Single-cell RNA sequencing (scRNA-seq) has been applied for uncovering molecular programs and lineage progression of OA (Zhang et al., 2020c). For instance, Zhang et al. (2020c) identified the biomarkers and differentiation of chondrocytes in human OA using scRNA-seq analyses. Moreover, Ji et al. (2019) adopted scRNA-seq analyses to reveal chondrocyte taxonomy and mechanisms of human OA cartilage regeneration. Despite this, in-depth exploration should be conducted for clarifying the pathogenesis of OA. In this study, we identified differentially expressed genes (DEGs) in OA synovium and found that these DEGs mainly participated in inflammatory pathways based on transcriptome data. Moreover, we revealed immune cell subpopulations and their intercellular heterogeneity in OA synovial tissues by single-cell RNA sequencing (scRNA-seq) profiles. These findings might deepen our understanding on the transcriptomic diversities within synovial tissue.

## Materials and Methods

### OA Expression Profiles

Gene expression profiles of GSE55235 and GSE55457 were retrieved from the Gene Expression Omnibus (GEO) repository (https://www.ncbi.nlm.nih.gov/gds/) using the GEOquery package (version 3.6.1; https://www.r-project.org/). The GSE55235 dataset contained microarray expression profiling of 10 synovial tissues from healthy joints and 10 synovial tissues from osteoarthritic joints (Woetzel et al., 2014). The GSE55457 dataset contained expression profiling of 10 synovial tissue specimens from normal joints and 10 synovial tissue specimens from osteoarthritic joints (Woetzel et al., 2014). Above datasets were based on GPL96 platform, [HG-U133A] Affymetrix Human Genome U133A Array.

### Differential Expression Analyses

Raw CEL files were read by affy package (version 1.0) (Gautier et al., 2004). Microarray expression profiling was normalized with Robust MultiChip Analysis (RMA) method (Irizarry et al., 2003) and standardized by quantile. The expression value was then log2 converted. The Linear Models for Microarray Data (limma; version 1.0) package was applied for screening DEGs between OA and healthy synovial tissues with student’s t-test in the GSE55235 and GSE55457 datasets (Ritchie et al., 2015). False discovery rate (FDR) was determined for multiple comparisons with Benjamini and Hochberg method. Genes with FDR<0.05 and |fold-change|>1.5 were defined as DEGs. Hierarchical clustering analyses of DEGs were presented with the gplots package. DEGs in the two datasets were intersected to obtain common DEGs for OA.

### Functional Annotation Analysis

The biological functions and pathways of common DEGs were interpreted via the clusterProfiler package (version 1.0; http://bioconductor.org/packages/release/bioc/html/clusterProfiler.html) (Yu et al., 2012). Biological processes of gene ontology (GO) were functionally annotated for these DEGs. For understanding signaling pathways enriched by these DEGs, Kyoto encyclopedia of genes and genomes (KEGG) pathway enrichment analysis was employed. Terms with FDR<0.05 were considered significant enrichment. The top ten biological processes and the top 30 KEGG pathways were visualized.

### CIBERSORT Analysis

The gene expression profiles of the two datasets were integrated into one dataset. The CIBERSORT algorithm (version 1.0; http://cibersort.stanford.edu/) was applied to characterize immune cell compositions in each specimen based on normalized gene expression profiling (Newman et al., 2015). The specimens with *p* < 0.05 were screened and the proportions of 22 kinds of immune cells were determined, including B cells naïve, B cells memory, plasma cells, T cells CD8, T cells CD4 naïve, T cells CD4 memory resting, T cells CD4 memory activated, T cells follicular helper, T cells regulatory (Tregs), T cells gamma delta, NK cells resting, NK cells activated, monocytes, macrophages M0, macrophages M1, macrophages M2, dendritic cells resting, dendritic cells activated, mast cells resting, mast cells activated, eosinophils and neutrophils. Principal component analysis (PCA) was presented for determining the differences in the proportions of immune cells between OA and healthy synovial tissue specimens. The infiltration levels of immune cells between groups were compared using the vioplot package.

### ScRNA-Seq and Preprocessing

Single-cell transcriptomic data of 10,640 synoviocytes from 3 osteoarthritic synovial membrane samples were obtained from the GSE152805 dataset on the GPL20301 Illumina HiSeq 4000 (Homo sapiens) (Chou et al., 2020). The Seurat package (version 3.0; http://satijalab.org/seurat/) was employed for quality control (Butler et al., 2018). Cells with 200–5000 feature genes and the percent of transcripts of mitochondrial genes <10% were retained for further analysis. Expression data were normalized with the LogNormalize method. Briefly, based on the total expression levels, the gene expression value in each cell was normalized and then multiplied by a scale factor = 10,000, followed by log2 conversion. Gene variances were calculated via the FindVariableFeatures function. Each dataset returned the top 2000 highly variable genes, which were used for downstream analysis. Meanwhile, the top 10 genes with the highest variances were selected and visualized. Then, the data were scaled utilizing the ScaleData function, as follows: the expression of each gene was shifted so that the average expression between cells was 0 and the expression of each gene was scaled so that the difference between cells was 1. After scaling the data, PCA was presented for the 2000 highly variable genes with the VizDimReduction and DimPlot functions. The number of principal components (PCs) was determined with the elbow plot method.

### Cell Cluster

In PCA, a KNN graph was constructed based on Euclidean distance. The FindNeighbors function was applied to find their edge weights between any 2 cells. Cell cluster was analyzed based on the FindClusters function. Non-linear dimensionality reduction technology t-distributed statistical neighbor embedding (tSNE) was employed to cluster similar cells together in a low-dimensional space. Markers were identified for each cluster using the FindAllMarkers function, which were visualized with the DoHetmap, VlinPlot and FeaturePlot functions. Based on the markers of each cluster, cell type was annotated via the Cell Type Annotation of Single Cells (SingleR; version 1.0) (Aran et al., 2019).

### Pseudo-Time Analysis

Monocle algorithm (version 2.0) was applied for pseudo-time analysis (Qiu et al., 2017). Genes that were expressed in >5% of the cells were selected. The t-SNE was presented with reduce Dimension function and cells were clustered with cluster Cells function. Genes that were differentially expressed (p-value < 0.05) between clusters were selected as candidate genes with differential Gene Test function. Through the reduce Dimension function, based on the above candidate genes, dimensionality reduction analysis of the cells was caried out with DDRTree method. Then, cells were sorted and visualized according to single-cell trajectories using order Cells function.

### Ligand-Receptor Network Analysis

By CellPhoneDB (version 2.0) project (Efremova et al., 2020), the cell-cell communication was estimated based on the expression of receptors and ligands corresponding to each cell type. Ligand-receptor relationships between cells were then identified. Finally, ligand-receptor network was conducted *via* Cytoscape software (version 3.7.1) (Shannon et al., 2003).

### Identification of Differentially Expressed Marker Genes

The GSE55235 and GSE55457 datasets were integrated and batch effects were corrected. Differential expression analysis was carried out between OA and normal synovial tissues. Genes with adjusted *p* < 0.05 and |fold-change|>1.5 were considered differentially expressed. By overlapping marker genes in each cell type, differentially expressed marker genes were identified. Functional annotation analysis of above marker genes was performed.

### Cell Culture and Establishment of OA Cellular Models

Human chondrocytes C28/I2 (ATCC, United States) were grown in DMEM (Sigma, United States) as well as were incubated in a humidified environment with 10% fetal bovine serum containing 5% CO_2_ at 37°C. Chondrocytes were planted into a six-well plate and exposed to 10 ng/ml IL-1β (Sigma, United States) lasting 24 h to establish OA cellular models.

### Western Blotting

Chondrocytes were lysed with RIPA buffer (pH 8.0) and extracted protein was quantified with BCA Protein Quantitation Kit. Thereafter, protein was isolated on SDS-PAGE gels as well as transferred onto PVDF membrane. The membrane was sealed lasting 1 h utilizing PBST plus 5% BSA and incubated by specific primary antibodies at 4°C overnight, containing NR4A1 (1:500; #25851-1-AP; Proteintech, Wuhan, China), NR4A2 (1:500; #66878-1-Ig; Proteintech), GAPDH (1:1000; #60004-1-Ig; Proteintech), MMP3 (1:500; #17873-1-AP; Proteintech), MMP13 (1:1000; #18165-1-AP; Proteintech) and Collagen II (1:800; #28459-1-AP; Proteintech). Afterwards, the membrane was incubated by HRP-conjugated goat anti-rabbit secondary antibody (1:2000; #SA00001-2; Proteintech) lasting 1 h. Protein bands were visualized utilizing ECL kits.

### Transfection

Small interfering RNAs (siRNAs) of NR4A1 (si-NR4A1) and NR4A2 (si-NR4A2) were synthesized by GenePharma (Shanghai, China). IL-1β-induced chondrocytes were seeded onto a six-well plate (1×10^6^/well). When cell confluence reached 80–90%. In line with the manufacturer’s protocol, cell transfection was presented utilizing Lipofectamine 2000 Reagent (Invitrogen, United States). After 48 h, NR4A1 and NR4A2 expressions were verified with western blotting.

### Flow Cytometry

Chondrocyte apoptosis was determined utilizing Annexin V-FITC Apoptosis Detection kits (BD, United States) in accordance with the manufacturer’s protocol. Chondrocytes were collected and washed twice by PBS. Thereafter, chondrocytes were suspended through adding Annexin V, followed by incubation at 4°C lasting 15 min in the dark. Chondrocytes were incubated by propidium iodide lasting 5 min in the dark. Stained chondrocytes were tested utilizing flow cytometer (FACSCalibur; BD, United States).

### 5-Ethynyl-2′-Deoxyuridine (EdU) Staining

EdU cellular proliferation experiment was conducted. In brief, pre-warmed 2× EdU working solution (RiboBio, China) was added to the cell plate to make EdU 10 Μm concentration, followed by incubation lasting 2 h. Thereafter, chondrocytes were fixed by 4% paraformaldehyde as well as permeated by 0.3% Triton X-100 solution at room temperature lasting 15 min. DAPI (Sigma, United States) was then added to each well as well as incubated lasting 10 min. Images were captured under a Nikon A1Si Laser Scanning Confocal Microscope (Nikon, Japan).

### Statistical Analyses

Data were displayed as the mean ± SD, and statistical analyses were carried out using Student’s t test or one-way ANOVA for multiple comparisons. *p* < 0.05 was statistically significant. All statistical analyses were implemented utilizing R software (version 3.6.1; https://www.r-project.org) and GraphPad Prism (version 8.0.1).

## Results

### Screening DEGs in OA Synovial Tissues

**FIGURE 1:**
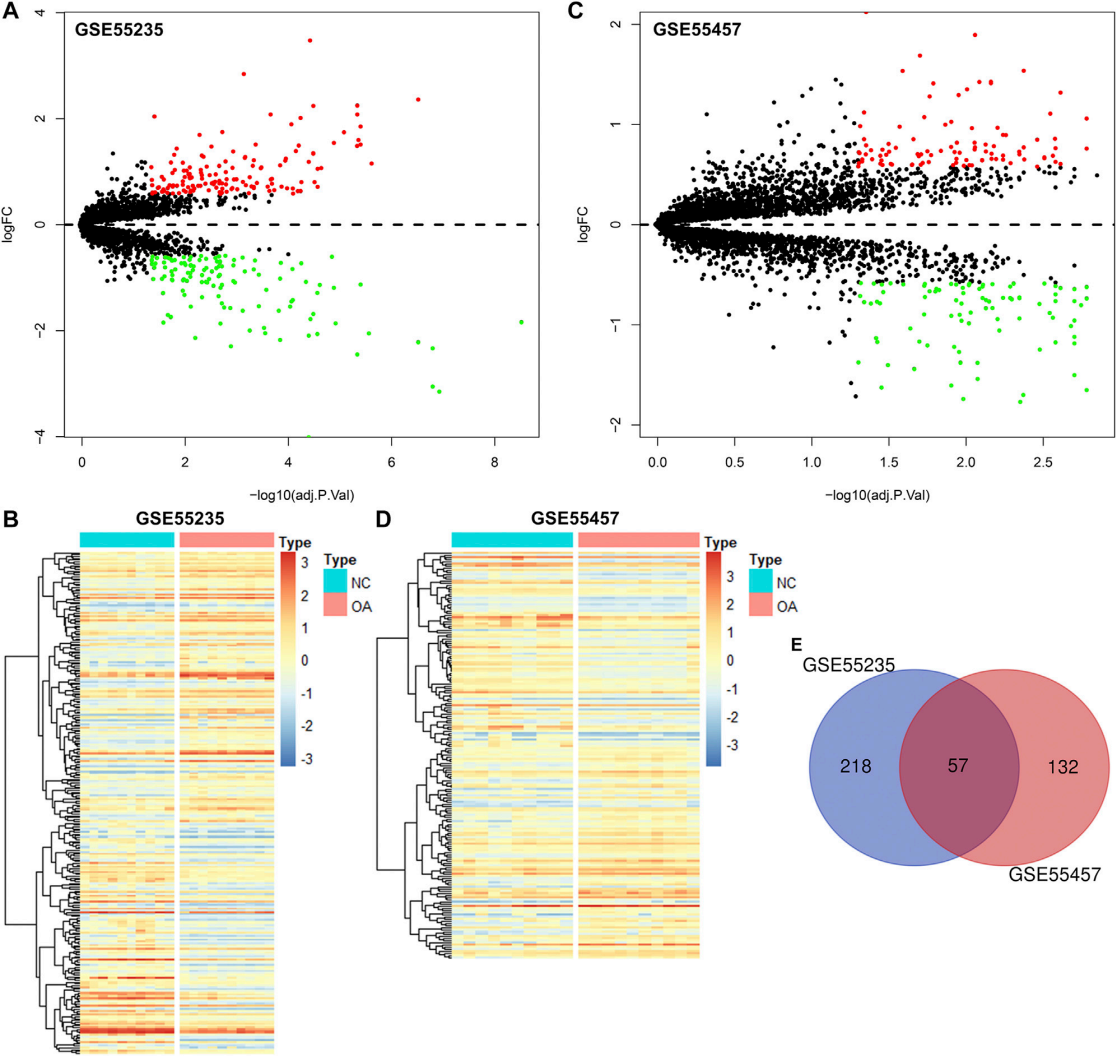
Identification of DEGs for OA synovial tissues. **(A)** Volcano diagram of DEGs in OA compared to healthy synovial tissues in the GSE55235 dataset. Red: up-regulation and green: down-regulation. X-axis: −log10(adjusted p-value) and y-axis: log[fold-change (FC)]. **(B)** Heatmap of DEGs in OA (red) than healthy (blue) synovial samples in the GSE55235 dataset. N: normal and O: osteoarthritis. **(C,D)** Volcano and heatmap plots of DEGs in OA than healthy synovial tissue specimens in the GSE55457 dataset. **(E)** Venn diagram of common DEGs in the GSE55235 and GSE55457 datasets.

The gene expression profiles of OA and healthy synovial tissue specimens were obtained from two microarray datasets GSE55235 and GSE55457. With the criteria of FDR<0.05 and |fold-change|>1.5, 148 genes were up-regulated and 127 genes were down-regulated in OA compared to healthy synovial tissues in the GSE55235 dataset (Figures 1A,B; Supplementary Table S1). Meanwhile, there were 98 up- and 91 down-regulated genes in OA synovial tissues in the GSE55457 dataset (Figures 1C,D; Supplementary Table S2). To obtain genes closely related to OA, 57 common DEGs were taken intersection in the two datasets, as follows: EMP1, PTN, ITGA5, IL10RA, FOSL2, EPHA3, ZFP36, STC1, TRIL, CHD1, HTR2B, ACOT13, SFPQ, CCNL1, NAP1L3, B3GALNT1, GLT8D2, CASP1, GTF2H5, EML1, PTGS2, YBX3, HPGDS, THBS4, SMCO4, NR4A1, FKBP5, TLR7, DDX3Y, NFKBIA, JUNB, PTGS1, MYL6B, SLC2A3, SAMSN1, XIST, LONRF1, TBL1XR1, VEGFA, SLC5A3, EFNB2, MYC, JUN, PNMAL1, PSMB10, KCNN4, PRSS23, NTAN1, TM6SF1, MAFF, TNFAIP3, MCL1, TMEM230, HLA-DQB1, GADD45B, IL11RA and RLF (Figure 1E; Table 1).

**TABLE 1:** 57 common DEGs for OA in the GSE55235 and GSE55457 datasets.

Genes	GSE55235			GSE55457		
	logFC	Average expression	FDR	logFC	Average expression	FDR
EMP1	−1.53804	7.887924	0.000374	−0.78463	8.94808	0.03614
PTN	1.693315	7.277286	0.005267	1.893238	8.970194	0.00875
ITGA5	−0.97312	9.63615	0.002258	−0.6721	9.928528	0.034423
IL10RA	0.818728	8.861961	0.011105	0.608606	10.18945	0.035901
FOSL2	−1.8408	7.840627	3.05E-09	−0.89903	8.173713	0.01102
EPHA3	1.206541	7.104439	0.000115	0.59649	7.47898	0.034815
ZFP36	−0.95171	11.72249	0.004728	−1.21946	11.63389	0.011941
STC1	−1.08969	7.041035	0.001088	−0.78669	7.371746	0.010717
TRIL	0.86547	7.001597	0.000209	1.074511	8.095606	0.018674
CHD1	−1.18988	7.337441	0.002073	−0.76874	8.492246	0.037772
HTR2B	0.812313	6.223356	0.002623	1.409481	7.419398	0.016303
ACOT13	0.707303	8.165143	0.032222	0.731063	9.539617	0.007443
SFPQ	−0.72694	8.819669	0.011862	−0.7211	9.029602	0.018476
CCNL1	−1.86073	9.960175	1.21E-05	−1.37882	10.10339	0.008427
NAP1L3	1.262345	6.776525	0.000698	1.536417	7.96821	0.00423
B3GALNT1	0.58516	5.584658	8.12E-05	0.896323	6.929353	0.005763
GLT8D2	1.1194	9.138441	0.000145	0.81933	10.14097	0.008812
CASP1	0.763415	8.585085	0.00281	0.601182	9.847556	0.030216
GTF2H5	0.613028	7.943939	0.00215	0.728144	9.254297	0.031534
EML1	1.036114	8.055376	0.004998	0.766664	8.773258	0.028549
PTGS2	−2.29285	6.570394	0.001299	−1.37402	6.275423	0.049855
YBX3	−1.07976	11.86993	4.57E-05	−0.58437	11.58165	0.030883
HPGDS	1.486339	7.993088	4.66E-06	0.714001	9.626692	0.049047
THBS4	1.18456	12.59617	4.03E-05	0.984282	13.37709	0.048564
SMCO4	0.891187	8.376471	0.00821	0.596613	9.972678	0.022444
NR4A1	−1.48429	9.384951	0.001946	−1.74013	9.246797	0.010422
FKBP5	−1.74073	7.799668	0.018898	−1.60501	7.744747	0.012483
TLR7	1.544719	7.293059	1.31E-05	1.425065	8.339314	0.008208
DDX3Y	−1.70292	6.52741	0.021566	−1.6252	6.637424	0.035333
NFKBIA	−2.21872	12.16799	3.05E-07	−0.73283	12.20779	0.007824
JUNB	−1.62412	11.10396	0.000153	−1.1676	10.63951	0.020095
PTGS1	0.71453	8.367016	0.00047	0.770843	9.246738	0.00876
MYL6B	0.718007	8.68208	0.000136	0.742958	9.79811	0.041344
SLC2A3	−2.04758	8.119309	0.000281	−1.27151	7.961026	0.011104
SAMSN1	1.084399	7.039047	0.045119	0.722377	7.927955	0.049589
XIST	2.041557	7.505913	0.039329	2.121796	7.405856	0.044492
LONRF1	−0.67573	6.833971	0.000445	−0.59838	6.96574	0.011596
TBL1XR1	1.109469	6.843264	0.0022	0.70277	7.745588	0.04465
VEGFA	−1.32466	7.235039	0.010343	−1.1704	8.023921	0.037619
SLC5A3	1.494343	7.703155	6.30E-05	1.027719	8.293338	0.012483
EFNB2	−0.80397	8.500263	0.024374	−0.71185	8.572233	0.048564
MYC	−2.44781	9.995651	4.61E-06	−1.37812	9.492429	0.010881
JUN	−2.17282	9.730232	0.000145	−1.1676	10.63951	0.020095
PNMAL1	0.781614	6.083028	0.003034	1.351997	7.56205	0.00985
PSMB10	0.857659	7.792863	0.001575	0.718192	9.405674	0.01102
KCNN4	0.649051	7.540138	0.003499	0.650437	8.451175	0.047106
PRSS23	1.511838	10.22883	3.99E-06	0.581612	11.49779	0.049969
NTAN1	0.719251	8.024924	0.017429	0.641965	9.534103	0.035368
TM6SF1	0.9808	8.36094	0.004922	1.06001	9.736704	0.001656
MAFF	−1.19152	8.353055	1.31E-05	−0.72953	8.253915	0.00576
TNFAIP3	−2.4492	9.204496	4.61E-06	−0.89588	9.24711	0.018711
MCL1	−0.81764	8.435161	0.003895	−0.88168	8.653155	0.002871
TMEM230	0.950574	9.583572	0.008609	0.848139	10.62885	0.003461
HLA-DQB1	1.473171	10.44126	0.003101	1.279768	11.72487	0.017225
GADD45B	−3.05288	10.60088	1.60E-07	−1.76873	10.30909	0.004456
IL11RA	1.053589	8.255707	2.72E-05	0.998688	8.779108	0.013644
RLF	−0.94581	7.440511	0.000374	−0.6129	8.383559	0.002634

### Functional Annotation Analysis of Common DEGs

**FIGURE 2:**
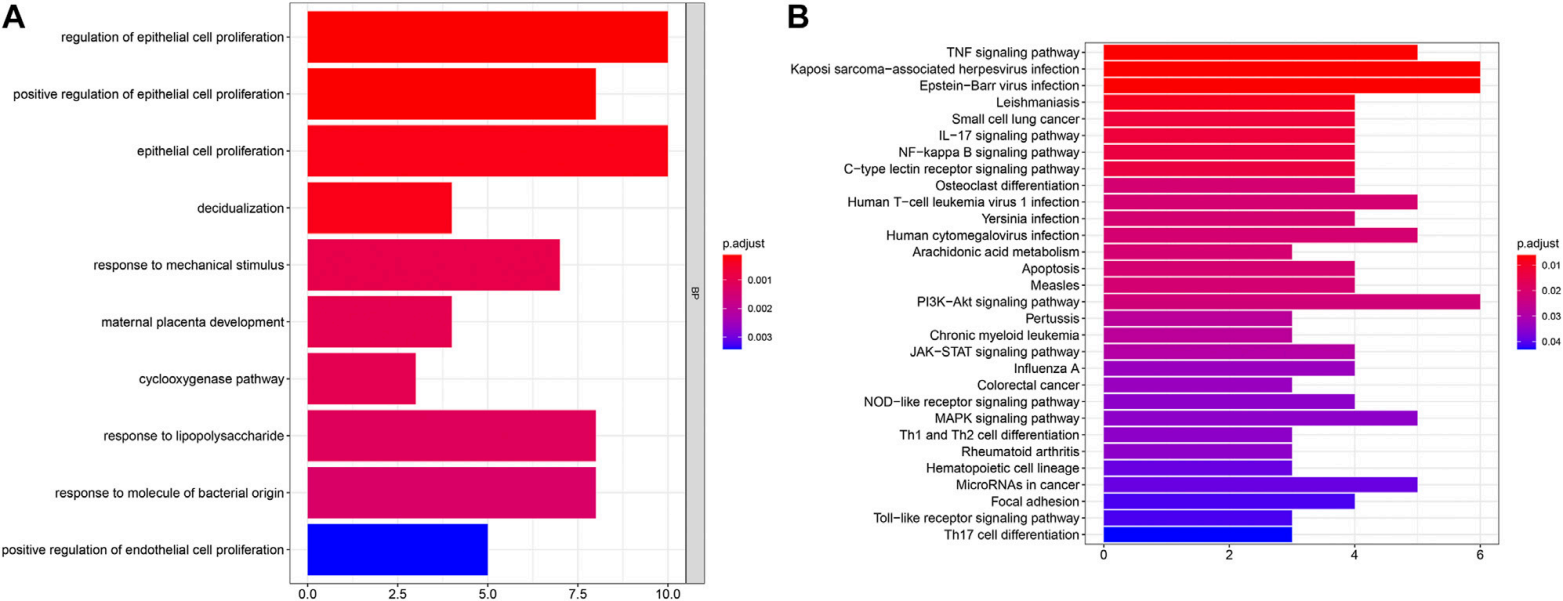
GO and pathway enrichment analysis of DEGs. **(A)** The top ten biological processes of common DEGs. X-axis represents the number of enriched DEGs and y-axis represents the biological process terms. The more the color tends to red, the smaller the adjusted p-value. **(B)** The top 30 KEGG pathways enriched by common DEGs.

To uncover biological functions and pathways of these 57 common DEGs, functional enrichment analyses were carried out. GO enrichment results showed that these DEGs were involved in regulating epithelial cell proliferation and response to mechanical stimulus, lipopolysaccharide and molecule of bacterial origin (Figure 2A; Table 2). Moreover, several key pathways were distinctly enriched by these DEGs such as TNF, IL-17, NF-kappa B, apoptosis, osteoclast differentiation, PI3K-Akt, JAK-STAT, Toll-like receptor and Th17 cell differentiation pathways (Figure 2B; Table 2).

**TABLE 2:** The detailed information of GO and pathway enrichment analysis results.

Description	GeneRatio	pvalue	p.adjust	qvalue	Count
Regulation of epithelial cell proliferation	10/55	1.34E-07	0.000131	9.66E-05	10
Positive regulation of epithelial cell proliferation	8/55	1.50E-07	0.000131	9.66E-05	8
Epithelial cell proliferation	10/55	4.77E-07	0.000279	0.000205	10
Decidualization	4/55	6.85E-07	0.000301	0.000221	4
Response to mechanical stimulus	7/55	2.65E-06	0.000929	0.000684	7
Maternal placenta development	4/55	3.30E-06	0.000965	0.00071	4
Cyclooxygenase pathway	3/55	3.93E-06	0.000985	0.000724	3
Response to lipopolysaccharide	8/55	5.17E-06	0.001135	0.000835	8
Response to molecule of bacterial origin	8/55	6.86E-06	0.001339	0.000985	8
Positive regulation of endothelial cell proliferation	5/55	1.95E-05	0.003416	0.002512	5
TNF signaling pathway	5/32	7.01E-05	0.006066	0.003865	5
Kaposi sarcoma-associated herpesvirus infection	6/32	9.31E-05	0.006066	0.003865	6
Epstein-Barr virus infection	6/32	0.00012	0.006066	0.003865	6
Leishmaniasis	4/32	0.000224	0.008529	0.005434	4
Small cell lung cancer	4/32	0.000445	0.012222	0.007787	4
IL-17 signaling pathway	4/32	0.000482	0.012222	0.007787	4
NF-kappa B signaling pathway	4/32	0.000708	0.013444	0.008565	4
C-type lectin receptor signaling pathway	4/32	0.000708	0.013444	0.008565	4
Osteoclast differentiation	4/32	0.001536	0.020844	0.01328	4
Human T-cell leukemia virus 1 infection	5/32	0.001552	0.020844	0.01328	5
*Yersinia* infection	4/32	0.001626	0.020844	0.01328	4
Human cytomegalovirus infection	5/32	0.00175	0.020844	0.01328	5
Arachidonic acid metabolism	3/32	0.00191	0.020844	0.01328	3
Apoptosis	4/32	0.00192	0.020844	0.01328	4
Measles	4/32	0.00208	0.021073	0.013426	4
PI3K-Akt signaling pathway	6/32	0.002341	0.022239	0.014169	6
Pertussis	3/32	0.003266	0.027581	0.017573	3
Chronic myeloid leukemia	3/32	0.003266	0.027581	0.017573	3
JAK-STAT signaling pathway	4/32	0.003622	0.02898	0.018464	4
Influenza A	4/32	0.004395	0.033402	0.021281	4
Colorectal cancer	3/32	0.004629	0.033506	0.021347	3
NOD-like receptor signaling pathway	4/32	0.005377	0.035025	0.022315	4
MAPK signaling pathway	5/32	0.00555	0.035025	0.022315	5
Th1 and Th2 cell differentiation	3/32	0.00559	0.035025	0.022315	3
Rheumatoid arthritis	3/32	0.005761	0.035025	0.022315	3
Hematopoietic cell lineage	3/32	0.006852	0.039008	0.024853	3
MicroRNAs in cancer	5/32	0.006929	0.039008	0.024853	5
Focal adhesion	4/32	0.007763	0.041143	0.026213	4
Toll-like receptor signaling pathway	3/32	0.00785	0.041143	0.026213	3
Th17 cell differentiation	3/32	0.008487	0.043003	0.027398	3
Thyroid cancer	2/32	0.009294	0.045569	0.029033	2
Toxoplasmosis	3/32	0.009617	0.04568	0.029103	3
Serotonergic synapse	3/32	0.010335	0.047602	0.030328	3

### Landscape in Immune Infiltration Between OA and Healthy Synovial Tissues

**FIGURE 3:**
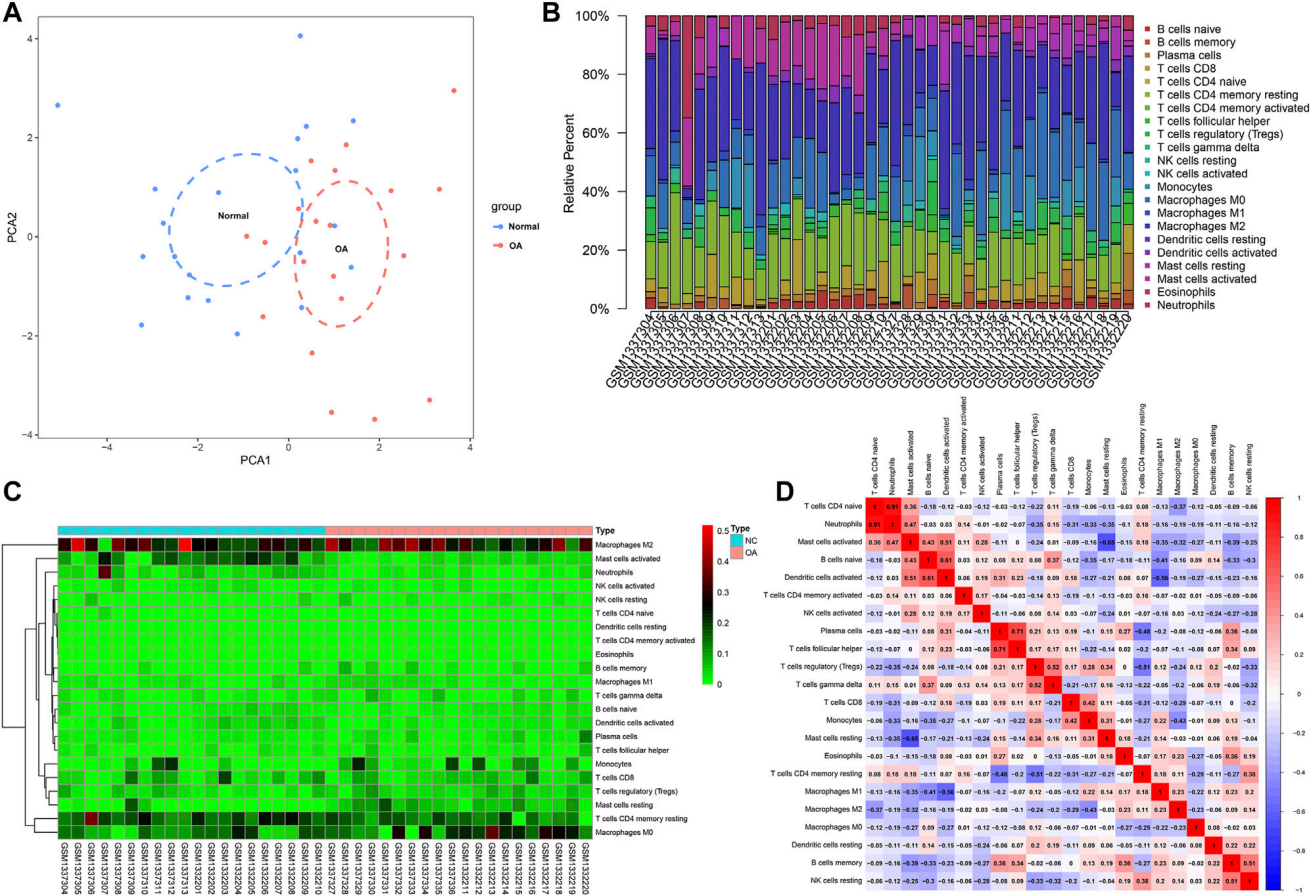
Landscape in immune infiltration between OA and healthy synovial tissue specimens from the GSE55235 and GSE55457 datasets. **(A)** PCA of synovial tissues based on immune cell infiltration levels. The first two principal components which decipher the most of the data variation. **(B)** The relative percent of 22 kinds of immune cells in each specimen. **(C)** Heatmap of the infiltration levels of immune cells in OA (red) and normal (blue) synovial tissues. **(D)** Positive (red) and negative correlations between different immune cells in synovial specimens. Correlation coefficient is marked in each box.

We employed the CIBERSORT algorithm to evaluate the immune cell infiltrations in OA and healthy synovial tissues from the integrated GSE55235 and GSE55457 datasets. Our PCA results demonstrated that the infiltration levels of immune cell compositions from OA and healthy synovial tissues exhibited significant group-bias cluster as well as personal disparity (Figure 3A). As shown in Figure 3B, we summarized the relative percent of different immune cells in each sample. Heatmap depicted that the infiltration levels of macrophages M2 were relatively high both in OA and healthy synovial tissue samples (Figure 3C). We further found that there were distinct correlations between immune cells (Figure 3D). Especially, T cells CD4 naïve were highly correlated to neutrophils (*r* = 0.91).

### Differences in Immune Infiltration Between OA and Healthy Synovial Tissues

**FIGURE 4:**
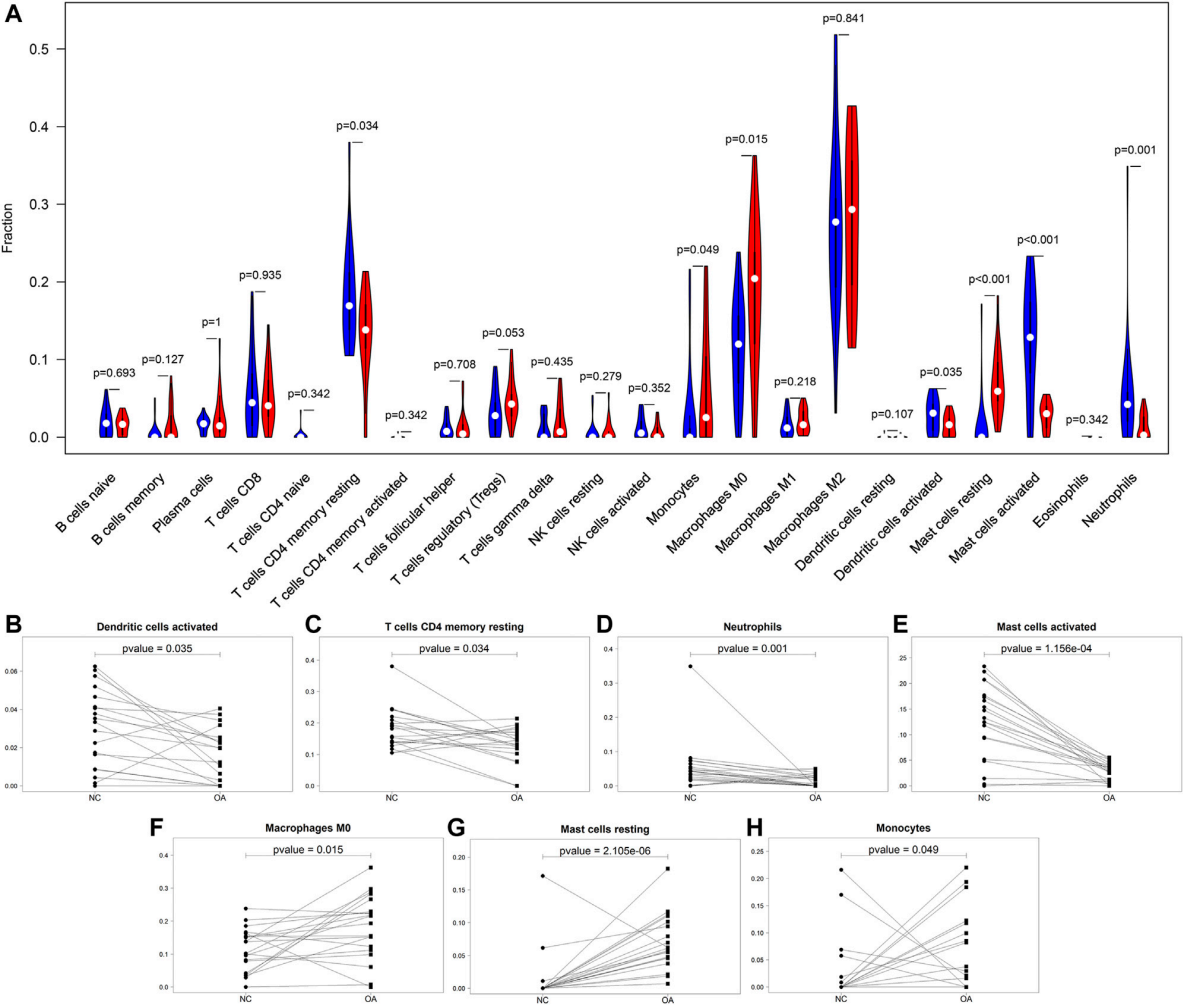
Comparisons of immune infiltration between OA and healthy synovial tissues. **(A)** Violin plots of the infiltration levels of immune cells in OA (red) and healthy (blue) synovial tissue specimens. Scatter plots for the significant differences in infiltration levels of **(B)** dendritic cells activated, **(C)** T cells CD4 memory resting, **(D)** neutrophils, **(E)** mast cells activated, **(F)** macrophages M0, **(G)** mast cells resting and **(H)** monocytes in OA and healthy synovial tissues.

This study compared the differences in infiltration levels of immune cells between OA and healthy synovial tissues from the GSE55235 and GSE55457 datasets with the CIBERSORT method (Figure 4A). Lower infiltration levels of dendritic cells activated (*p* = 0.035), T cells CD4 memory resting (*p* = 0.034), neutrophils (*p* = 0.001) and mast cells activated (*p* = 1.156e-04) were found in OA synovial tissues compared to healthy synovial tissues (Figures 4B–E). Oppositely, there were higher levels of macrophages M0 (*p* = 0.015), mast cells resting (*p* = 2.105e-06) and monocytes (*p* = 0.049) in OA than control synovial tissue specimens (Figures 4F–H).

### Quality Control of scRNA-Seq of Osteoarthritic Synoviocytes

**FIGURE 5:**
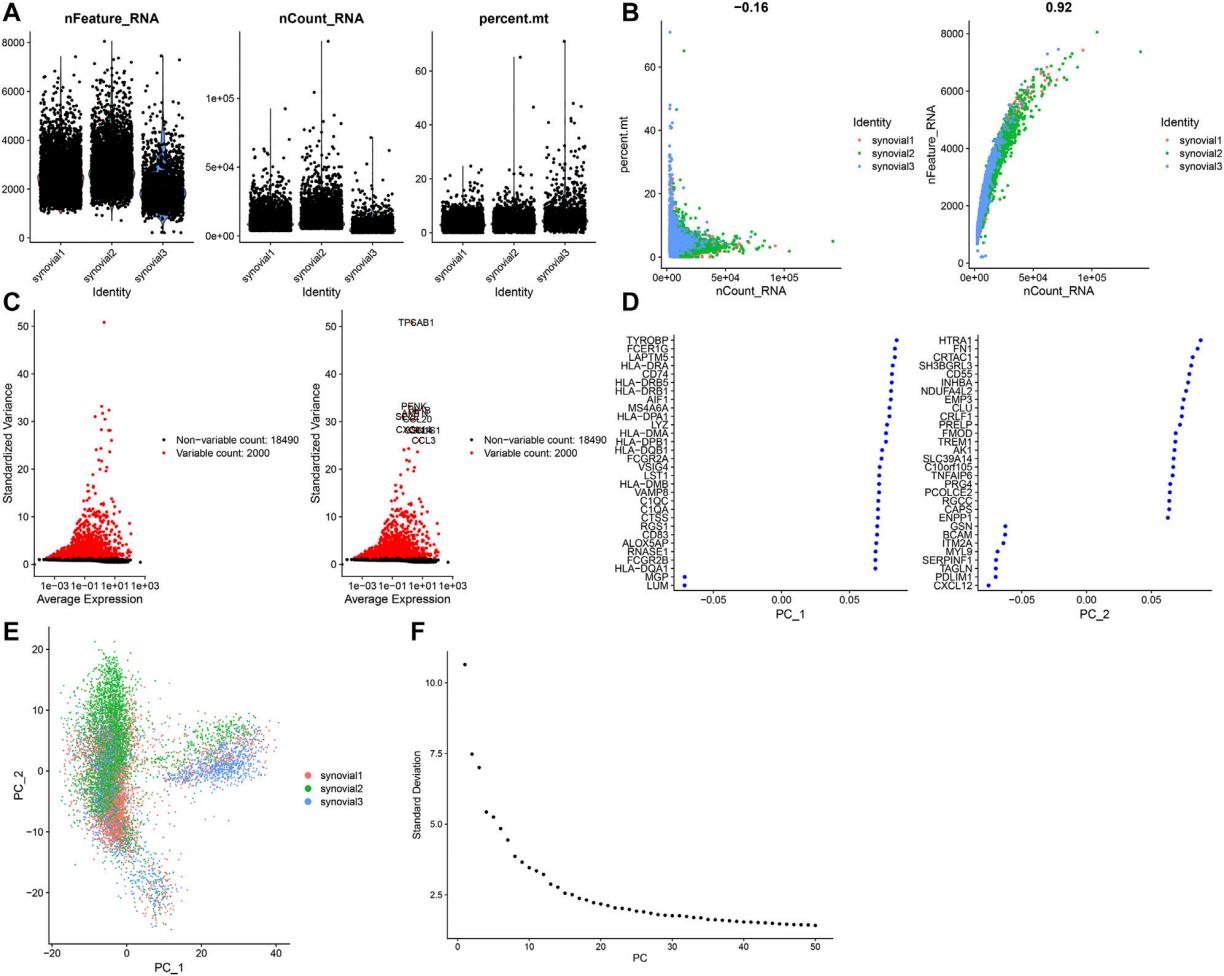
Quality control of scRNA-seq of osteoarthritic synoviocytes in the GSE152805 dataset. **(A)** Violin plots of the number of feature genes, the count of total genes and the percent of mitochondrial genes after filtration. **(B)** Scatter plots of correlations of the count of total genes with the percent of mitochondrial genes and the number of feature genes. **(C)** The top 2000 highly variable genes and visualization of the top ten genes. **(D,E)** Visualization of dimensionality reduction results of the scaling data. **(F)** Elbow plot for identifying the optimal number of PCs.

This study analyzed the scRNA-seq data of osteoarthritic synoviocytes from the GSE152805 dataset. We detected the genes in each cell and removed low-quality cells or empty droplets with very few genes <500 and cells with doublets or multiplets with high gene counts >2000. In addition, we filtered out the cells with percent of the mitochondrial genome >5%. After filtration, we visualized the number of feature genes and the count of genes as well as the percent of mitochondrial genes in OA synoviocytes (Figure 5A). Also, there was very low correlation between the count of genes and the percent of mitochondrial genes (Figure 5B). The count of genes was highly positively correlated to the number of feature genes. After filtration, the data were normalized with the LogNormalize method and the top 2000 highly variable genes were identified in OA synoviocytes for downstream analysis (Figure 5C). The top ten genes were as follows: TPSAB1, PENK, AMTN, IL1B, CCL4, CCL20, SELE, CXCL14, CCL3, and CSN1S1. After dimensionality reduction analysis, we visualized the highly variable genes and OA synoviocytes in the top two PCs (Figures 5D,E). With the elbow plot method, the top 30 PCs were determined for further analysis (Figure 5F).

### scRNA-Seq Uncovers Six Cell Types for OA Synoviocytes

**FIGURE 6:**
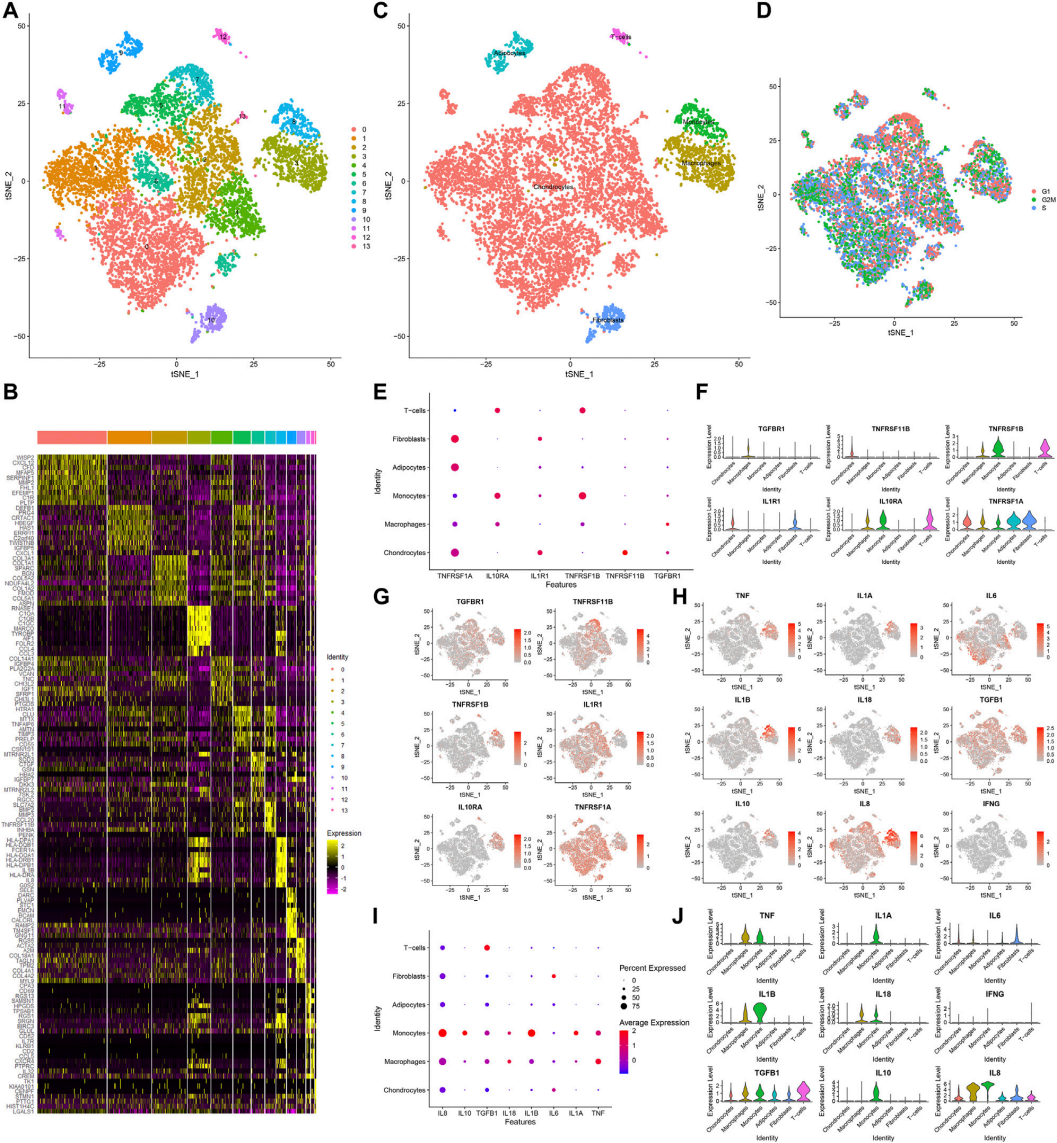
Identification of cell clusters and marker genes in OA synoviocytes. **(A)** Visualization of cell clusters marked by different colors for OA synoviocytes by t-SNE method. **(B)** Heat map of the scaled expressions of the top ten marker-genes for each cell cluster. **(C)** Annotation of t-SNE cell types for OA synoviocytes. **(D)** Cell cycle (G1, G2M, S) of OA synoviocytes on the t-SNE map. **(E–G)** Visualization of the expression levels of cytokine receptor marker genes for each cluster. **(H–J)** Visualization of the expression levels of inflammatory cytokine marker genes for each cluster.

Totally, 10,154 OA synoviocytes were clustered into 14 cell populations via the t-SNE method (Figure 6A). As shown in Figure 6B, we visualized the top 10 differentially expressed marker genes for each cell cluster. With the SingleR package, five cell types were annotated, as follows (from most to least): chondrocytes, macrophages, monocytes, fibroblasts, adipocytes and T cells (Figure 6C). Also, the cell cycle distribution of each cell cluster was shown (Figure 6D). Secreted inflammatory cytokines and their receptors are considered as key mediators for OA progression (Kapoor et al., 2011). Here, we analyzed the expression distribution of cytokine receptors in cell types. As a result, TGFBR1 was mainly expressed in macrophages and TNFRSF11B was mainly expressed in chondrocytes (Figures 6E–G). Meanwhile, TNFRSF1B was primarily distributed in monocytes, followed by T cells and macrophages. IL1R1 was mainly expressed in fibroblasts and chondrocytes, while IL10RA was primarily distributed in T cells, followed by monocytes and macrophages. Also, TNFRSF1A was expressed in fibroblasts, adipocytes, chondrocytes, macrophages and monocytes. This study further visualized the distribution of secreted inflammatory cytokines in each cell type (Figures 6H–J). TNF and IL-18 were expressed in macrophages and monocytes as well as IL1A and IL10 were only expressed in monocytes. IL-6 was primarily expressed in fibroblasts and IL1B was mainly distributed in monocytes, followed by macrophages. TGFB1 was mainly expressed in T cells, followed by monocytes, macrophages, adipocytes, fibroblasts and chondrocytes. Meanwhile, IL8 was primarily distributed in macrophages, followed by monocytes, fibroblasts, T cells, adipocytes and chondrocytes.

### Identification of Five Immune Cell Subpopulations and Associated Marker Genes for OA Synoviocytes

**FIGURE 7:**
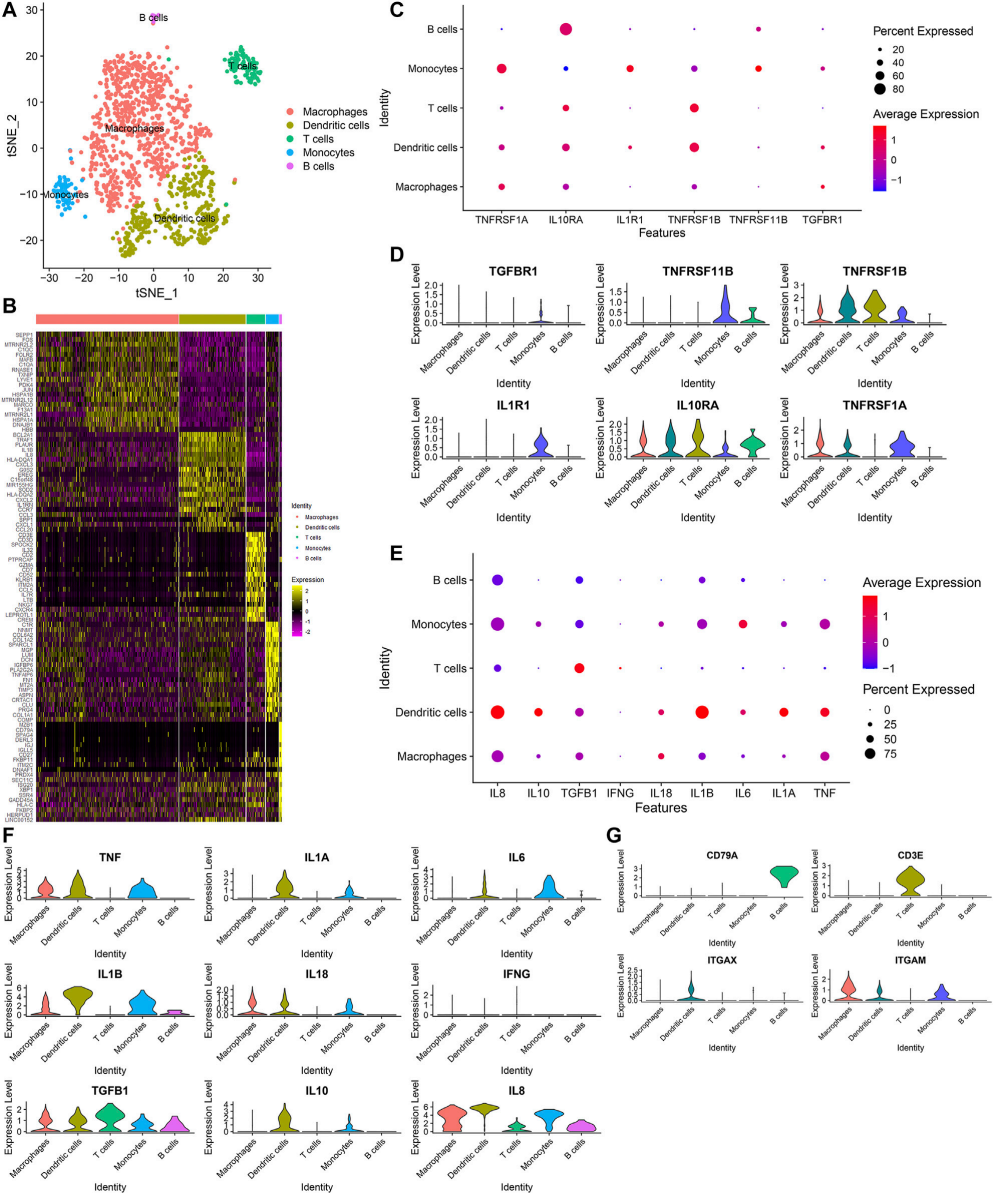
Identification of five immune cell subpopulations and associated marker genes for OA synoviocytes. **(A)** Visualization of five cell subpopulations with unique colors. **(B)** Heatmap for the scaled expression of the top 20 marker genes for each cell subpopulation. **(C,D)** Visualization of the expression levels of cytokine receptor marker genes for each cell subpopulation. **(E,F)** Visualization of the expression levels of inflammatory cytokine marker genes for each subpopulation. **(G)** Visualization of the expression levels of surface markers for each subpopulation.

Next, we further annotated the immune cell subpopulations of macrophages, monocytes and T cells across 1353 OA synoviocytes via the SingleR package. As a result, five cell subpopulations were obtained, including macrophages, dendritic cells, T cells, monocytes and B cells (from most to least; Figure 7A). The top 20 marker genes for each cell subpopulation were visualized, as shown in Figure 7B. The expression distributions of cytokine receptors were detected in each cell subpopulation, as follows: TGFBR1 (monocytes), TNFRSF11B (monocytes and B cells), TNFRSF1B (dendritic cells, T cells, monocytes and macrophages), IL1R1 (monocytes), IL10RA (T cells, monocytes, dendritic cells, macrophages, B cells and monocytes) and TNFRSF1A (monocytes, macrophages and dendritic cells; Figures 7C,D). The expression of inflammatory cytokines was shown in each cell subpopulation, as follows: TNF (dendritic cells, monocytes and macrophages), IL1A (dendritic cells and monocytes), IL6 (monocytes and dendritic cells), IL1B (dendritic cells, monocytes, macrophages and B cells), IL18 (macrophages, dendritic cells and monocytes), TGFB1 (T cells, dendritic cells, monocytes, macrophages and B cells), IL10 (dendritic cells and monocytes) and IL8 (macrophages, dendritic cells, monocytes, B cells and T cells; Figures 7E,F). Also, we found that CD79A was mainly expressed in B cells, CD3E was mainly expressed in T cells and ITGAX was mainly expressed in dendritic cells (Figure 7G). Meanwhile, ITGAM was mainly expressed in macrophages, dendritic cells and monocytes.

### Pseudo-Time Analysis of Osteoarthritic Synoviocytes

**FIGURE 8:**
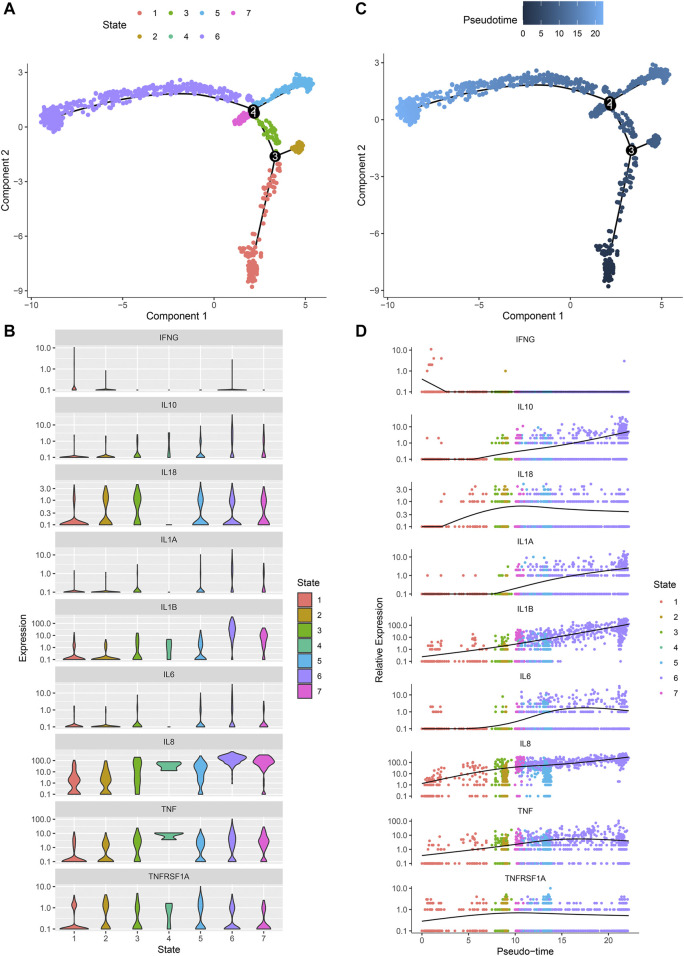
Pseudo-time analysis of osteoarthritic synoviocytes by Monocle 2 algorithm. **(A)** Distribution of osteoarthritic synoviocytes in 7 different states. **(B)** The mRNA expression of inflammatory cytokines and cytokine receptor in each state. **(C)** Distribution of osteoarthritic synoviocytes according to pseudo-time value. The darker the color, the larger the pseudo-time value. **(D)** Genes that dynamically change with pseudo-time value.

Monocle 2 algorithm was used to reshape the change process of cells over time by constructing the trajectory of changes between cells. Figure 8A showed the distribution of osteoarthritic synoviocytes in 7 different states. As shown in Figure 8B, we observed the heterogeneity in the mRNA expression of inflammatory cytokines (IFNG, IL10, IL18, IL1A, IL1B, IL6, IL8, and TNF) and cytokine receptor (TNFRSF1A) among states. The distribution of osteoarthritic synoviocytes according to pseudo-time value was shown in Figure 8C. The scatter plot demonstrated the dynamic expression of above genes over the pseudo-time value (Figure 8D). As the pseudo-time value increased, the mRNA expression of IFNG gradually decreased while the expression of IL10, IL18, IL1A, IL1B, IL6, IL8, TNF, and TNFRSF1A gradually increased. Above findings revealed the developmental characteristics of osteoarthritic synoviocytes.

### Ligand-Receptor Network

**FIGURE 9:**
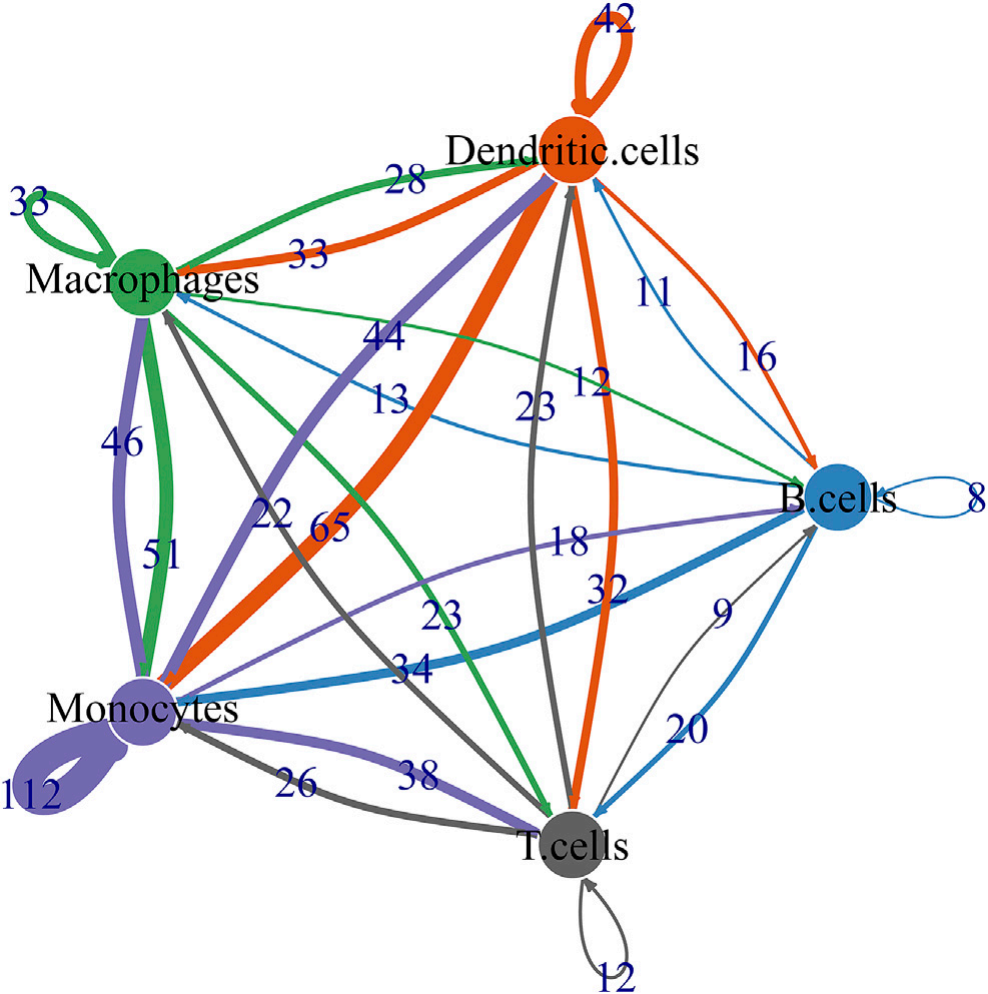
Ligand-receptor network based on B cells, dendritic cells, macrophages, monocytes, and T cells.

By CellPhoneDB v2.0, this study identified receptor-ligand relationships between five cell types. Figure 9 showed that the close interactions between cell types according to receptor-ligand interactions. The arrow pointed to the recipient cell. The width of the line represented the number of ligand-receptor relationships.

### Biological Functions of Differentially Expressed Marker Genes

**FIGURE 10:**
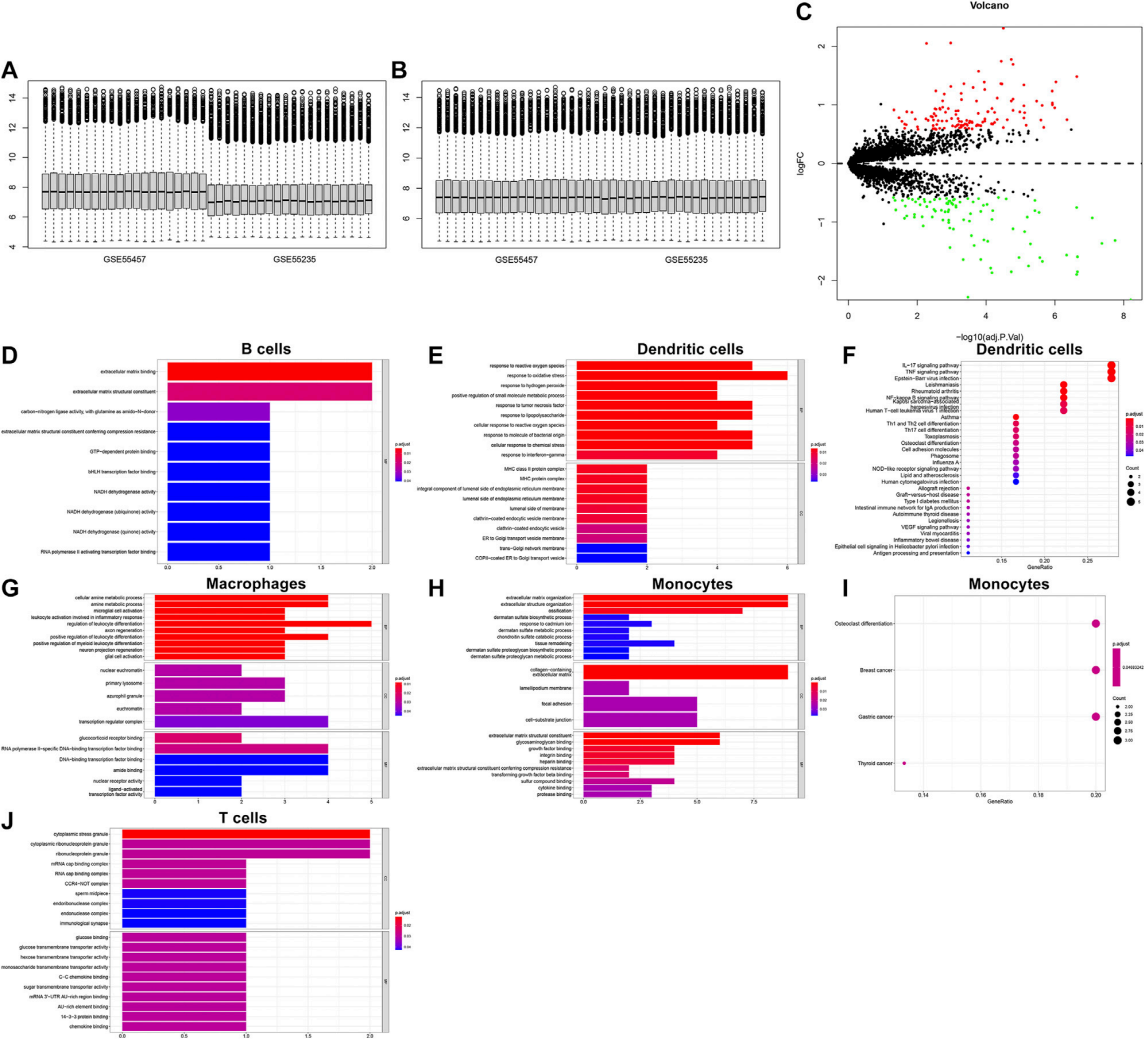
Biological functions of differentially expressed marker genes in five immune cells. **(A,B)** Before and after batch effects of the integrated GSE55235 and GSE55457 datasets. **(C)** Volcano plots of differentially expressed genes between OA and normal synovial tissues. **(D–J)** Functional annotation analysis of differentially expressed marker genes in **(D)** B cells, **(E,F)** dendritic cells, **(G)** macrophages, **(H,I)** monocytes, and **(J)** T cells.

We integrated the GSE55235 and GSE55457 datasets and removed batch effects (Figures 10A,B). With the criteria of FDR<0.05 and |fold-change|>1.5, we identified 121 up-regulated genes and 91 down-regulated genes in OA compared with normal synovial tissues (Figure 10C). After overlapping marker genes of each cell type, we finally identified 6 differentially expressed marker genes in B cells (ASNS, NDUFA3, SPARC, GCHFR, BHLHE41, and BGN), 22 differentially expressed marker genes in dendritic cells (CXCL3, FOSL2, MAP3K8, FCER1A, PTGS2, OLR1, KDM6B, PFKFB3, NFKBIA, GCH1, SAMSN1, HLA-DPB1, VEGFA, NR4A3, KCNN4, PMAIP1, MAFF, TNFAIP3, FOSL1, HLA-DQB1, CD58, and GLRX), 27 differentially expressed marker genes in macrophages (VAMP8, NR4A2, DNAJB1, HNMT, TREM2, NR4A1, NTAN1, GRN, TMEM230, MNDA, KLF4, EMP1, CRIP1, SLC7A7, CEBPD, GDE1, NCF1, HPGDS, CD83, FKBP5, KLF10, FOLR2, SGMS1, JUN, TMEM176B, GADD45B, and FRMD4B), 35 differentially expressed marker genes in monocytes (FERMT2, DPT, FAP, PCOLCE, PTGDS, GLT8D2, ANKH, TMCO3, THBS4, DDX3Y, JUNB, TNFRSF11B, PTPRG, PRSS23, IFI27, VCAN, GSN, TGFBR3, ANGPTL2, SSPN, THY1, PPIC, SPARC, TPPP3, FZD1, MYL6B, MYC, MT1X, CRISPLD2, BGN, COL1A2, IL1R1, LTBP3, APOC1, and GADD45B), and 9 differentially expressed marker genes in T cells (CD37, SLC2A3, SAMSN1, CD52, ZFP36, ELF1, PRRC2C, MT1X, and SCAF11). Functional annotation analysis was used for investigating their biological functions. We observed that differentially expressed marker genes in B cells were mainly involved in modulating the molecular functions of extracellular matrix (Figure 10D). Differentially expressed marker genes in dendritic cells markedly participated in immune-related processes and pathways (Figures 10E,F). In Figure 10G, differentially expressed marker genes in macrophages were mainly involved in modulating inflammatory response and leukocyte differentiation. Differentially expressed marker genes in monocytes primarily participated in osteoclast differentiation (Figures 10H,I). In Figure 10J, differentially expressed marker genes in T cells were mainly involved in regulating cytoplasmic stress granule.

### Silencing NR4A1 and NR4A2 Relieves IL-1β-Induced Chondrocyte Damage

**FIGURE 11:**
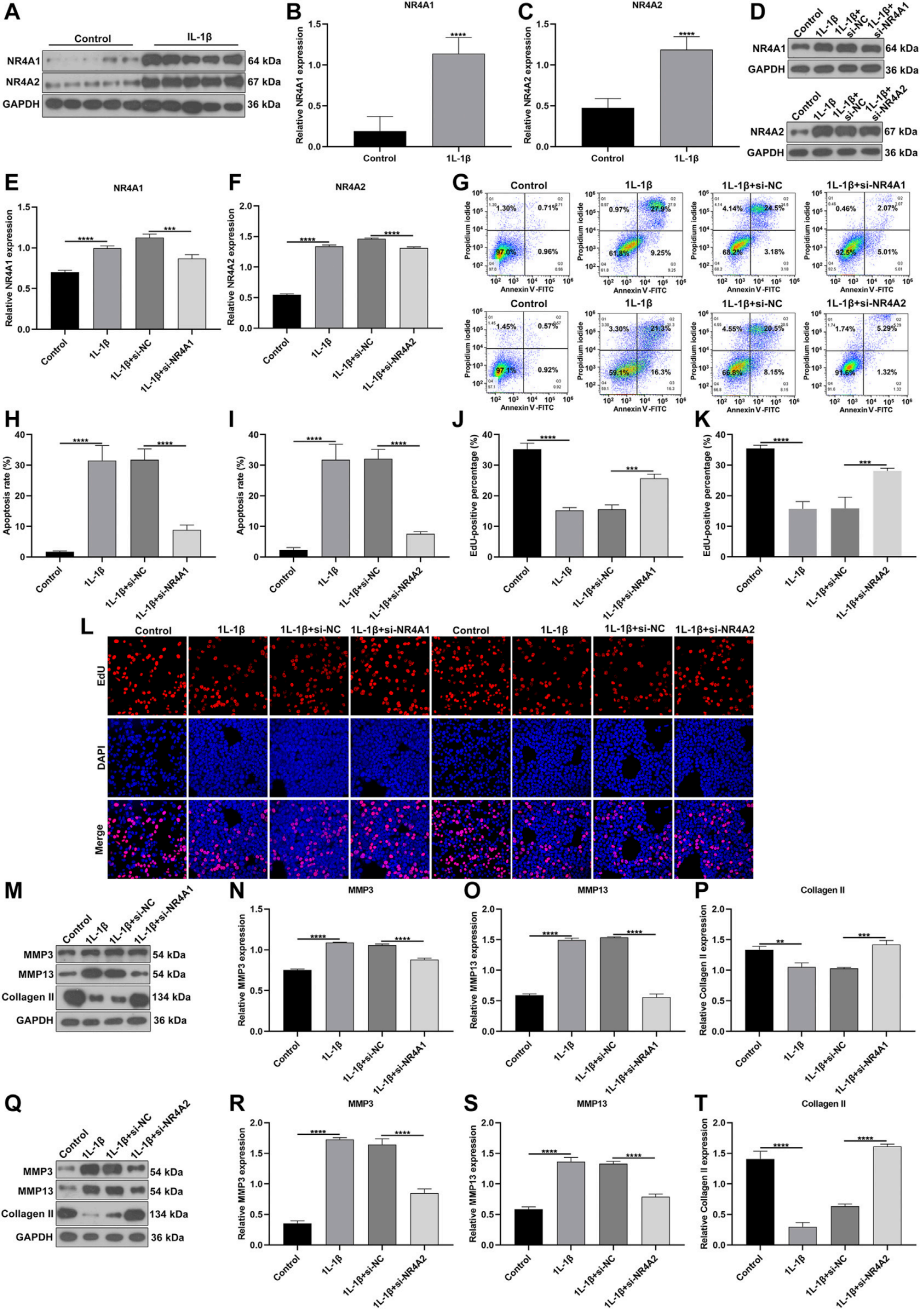
Silencing NR4A1 and NR4A2 relieves IL-1β-induced chondrocyte damage. **(A–C)** Western blotting of the expressions of NR4A1 and NR4A2 in IL-1β-induced chondrocytes. **(D–F)** Western blotting of the expressions of NR4A1 and NR4A2 in IL-1β-induced chondrocytes after silencing NR4A1 or NR4A2. **(G–I)** Flow cytometry detecting the apoptosis of IL-1β-induced chondrocytes with knockdown of NR4A1 and NR4A2. **(J–L)** EdU assay measuring the proliferation of IL-1β-induced chondrocytes with knockdown of NR4A1 and NR4A2. **(M–T)** Western blotting of the expressions of MMP3, MMP3 and Collagen II in IL-1β-induced chondrocytes under knockdown of NR4A1 and NR4A2. **p < 0.01; ***p < 0.001; ****p < 0.0001.

We further verified the biological functions of differentially expressed marker genes NR4A1 and NR4A2 in OA progression. IL-1β-induced OA chondrocyte models were constructed and our results confirmed the remarkable up-regulations of NR4A1 and NR4A2 in IL-1β-induced chondrocytes (Figures 11A–C). The expressions of NR4A1 and NR4A2 were then silenced by specific siRNAs in IL-1β-induced chondrocytes (Figures 11D–F). Flow cytometric analyses demonstrated that knockdown of NR4A1 and NR4A2 relieved IL-1β-induced chondrocyte apoptosis (Figures 11G–I). Additionally, silencing NR4A1 and NR4A2 enhanced proliferative capacity of IL-1β-induced chondrocyte apoptosis (Figures 11J–L). We also investigated that knockdown of NR4A1 and NR4A2 reduced the expressions of MMP3 and MMP3 as well as increased the expression of Collagen II in IL-1β-induced chondrocyte apoptosis (Figures 11M–T). Overall, silencing NR4A1 and NR4A2 relieved IL-1β-induced chondrocyte damage.

## Discussion

OA represents an age-related arthritis and the major chronic disability-related cause among elderly populations (Zhang et al., 2020a). OA is characterized by synovial inflammation, confirmed by magnetic resonance imaging and ultrasound (Conaghan et al., 2019). Here, based on microarray and scRNA-seq profiles, we comprehensively expounded immune cells and inflammatory mediators in OA synovium, which characterized the key features of OA pathogenesis. More importantly, our study depicted the intercellular heterogeneity of OA synovial tissues.

With the advance in RNA-seq and microarray techniques, more and more studies have revealed the molecular features of OA. Previous findings on gene expression profiles of OA synovial tissues have proposed several key genes as well as their enriched signaling pathways (Wang et al., 2020). Herein, this study identified 57 DEGs in OA compared to healthy synovial tissues. These genes were involved in several inflammatory pathways such as TNF, IL-17, NF-κB, Toll-like receptor and Th17 cell differentiation, indicating that they might be related to OA progression. Especially, as reported in a previous study, OA-related DEGs were also distinctly enriched in TNF pathway (Li et al., 2019). IL-17 regulates response to damage in OA by immunologically inducing senescence (Faust et al., 2020). NF-κB pathway affects synovial inflammation, which leads to the damage of joint cartilage (Choi et al., 2019). Moreover, Toll-like receptor may contribute to pathological alterations in OA joint tissue specimens (Miller et al., 2019). IL-17 mediates apoptosis in rheumatoid arthritis synoviocytes by activating autophagy (Kim et al., 2017). Therefore, alterations in gene expression may participate in inflammatory pathways of OA.

The inflammatory microenvironment of the OA synovium consists of immune cells and various inflammatory cytokines. The CIBERSORT algorithm confirmed that there were relatively high infiltration levels of macrophages in OA synovial tissues. Consistently, lower infiltration levels of dendritic cells activated, T cells CD4 memory resting, neutrophils and mast cells activated were detected in OA than healthy synovial tissues (Cai et al., 2020; Chen et al., 2020). On the contrary, there were higher infiltration levels of macrophages M0, mast cells resting and monocytes in OA compared to control synovial tissue specimens. These data were indicative that dysregulated immune cells in synovium might play key roles in OA pathogenesis. To further understand the roles of immune cells in the progression of OA, our scRNA-seq data demonstrated that OA may involve five immune cells including macrophages, dendritic cells, T cells, monocytes and B cells as well as associated effector cytokines and cytokine receptors in synoviocytes. The ligand-receptor network revealed the close crosstalk between cells. Among them, macrophages were most abundant immune cells. Consistently, Hsueh et al. (2021) found that macrophages were predominant in the OA synovial tissues, occupying 53% of total synoviocytes. Macrophages could product inflammatory cytokines including TNF, IL1B, IL18, TGFB1, and IL8. It has been confirmed that macrophages and macrophage-secreted mediators force the inflammatory and destructive responses in the OA synovial tissues (Bondeson et al., 2006). Therefore, macrophages may participate in the pathogenesis of OA. Inflammatory cytokines in the synovial membrane are also involved in the initiation and progression of OA (Li et al., 2020). For example, TNF-α induces the production of matrix metalloproteinases like MMP-13, ADAMTS-5 and ADAMTS-7 and activates the NF-κB pathway that can facilitate the proinflammatory functions of TNF-α (Zhao et al., 2019). Above data demonstrated that the immune cells, cytokines and receptors in synovial tissues might play critical roles in OA progression. Our *in vitro* experiments confirmed that silencing differentially expressed marker genes NR4A1 and NR4A2 relieved IL-1β-induced chondrocyte damage *in vitro* experiments. However, a few limitations should be pointed out. First, more scRNA-seq data should be verified for the intercellular heterogeneity of OA synovial tissues. Second, *in vivo* experiments should be carried out to verify the biological functions of NR4A1 and NR4A2 in OA progression.

## Conclusion

This study uncovered the transcriptomic diversities within OA synovial tissue. Our findings could deepen the understanding on the pathophysiology of OA and facilitate the progression of novel drugs against therapeutic targets.

## Data Availability

The original contributions presented in the study are included in the article/Supplementary Material, further inquiries can be directed to the corresponding author.

## Supplementary Material

Supplementary Table S1 DEGs of OA synovial tissues in the GSE55235 dataset.

Supplementary Table S2 DEGs of OA synovial tissues in the GSE55457 dataset.

## Abbreviations

DEGs, differently expressed genes; FC, fold-change; FDR, false discovery rate; GEO, gene expression omnibus; GO, gene ontology; KEGG, kyoto encyclopedia of genes and genomes; limma, linear models for microarray data; OA, osteoarthritis; PCA, principal component analysis; PCs, principal components; scRNA-seq, single-cell RNA sequencing; Tregs, T cells regulatory; t-SNE, t-distributed statistical neighbor embedding.
